# *In vitro* potency of xeruborbactam in combination with multiple β-lactam antibiotics in comparison with other β-lactam/β-lactamase inhibitor (BLI) combinations against carbapenem-resistant and extended-spectrum β-lactamase-producing *Enterobacterales*

**DOI:** 10.1128/aac.00440-23

**Published:** 2023-10-06

**Authors:** Olga Lomovskaya, Mariana Castanheira, Jill Lindley, Debora Rubio-Aparicio, Kirk Nelson, Ruslan Tsivkovski, Dongxu Sun, Maxim Totrov, Jeffery Loutit, Michael Dudley

**Affiliations:** 1 Qpex Biopharma, San Diego, California, USA; 2 JMI Laboratories, North Liberty, lowa, USA; 3 Molsoft, LLC, San Diego, California, USA; University of Fribourg, Fribourg, Switzerland

**Keywords:** xeruborbactam, taniborbactam, avibactam, relebactam, vaborbactam, carbapenemases, metallo-β-lactamases, CRE

## Abstract

Recently, several β-lactam (BL)/β-lactamase inhibitor (BLI) combinations have entered clinical testing or have been marketed for use, but limited direct comparative studies of their *in vitro* activity exist. Xeruborbactam (XER, also known as QPX7728), which is undergoing clinical development, is a cyclic boronate BLI with potent inhibitory activity against serine (serine β-lactamase) and metallo-β-lactamases (MBLs). The objectives of this study were (i) to compare the potency and spectrum of β-lactamase inhibition by various BLIs in biochemical assays using purified β-lactamases and in microbiological assays using the panel of laboratory strains expressing diverse serine and metallo-β-lactamases and (ii) to compare the *in vitro* potency of XER in combination with multiple β-lactam antibiotics to that of other BL/BLI combinations in head-to-head testing against recent isolates of carbapenem-resistant *Enterobacterales* (CRE). Minimal inhibitory concentrations (MICs) of XER combinations were tested with XER at fixed 4 or 8 µg/mL, and MIC testing was conducted in a blinded fashion using Clinical and Laboratory Standards Institute reference methods. Xeruborbactam and taniborbactam (TAN) were the only BLIs that inhibited clinically important MBLs. The spectrum of activity of xeruborbactam included several MBLs identified in *Enterobacterales,* e.g., and various IMP enzymes and NDM-9 that were not inhibited by taniborbactam. Xeruborbactam potency against the majority of purified β-lactamases was the highest in comparison with other BLIs. Meropenem-xeruborbactam (MEM-XER, fixed 8 µg/mL) was the most potent combination against MBL-negative CRE with MIC_90_ values of 0.125 µg/mL. MEM-XER and cefepime-taniborbactam (FEP-TAN) were the only BL/BLIs with activity against MBL-producing CREs; with MEM-XER (MIC_90_ of 1 µg/mL) being at least 16-fold more potent than FEP-TAN (MIC_90_ of 16 µg/mL). MEM-XER MIC values were ≤8 µg/mL for >90% of CRE, including both MBL-negative and MBL-positive isolates, with FEP-TAN MIC of >8 µg/mL. Xeruborbactam also significantly enhanced potency of other β-lactam antibiotics, including cefepime, ceftolozane, ceftriaxone, aztreonam, piperacillin, and ertapenem, against clinical isolates of *Enterobacterales* that carried various class A, class C, and class D extended-spectrum β-lactamases and carbapenem-resistant *Enterobacterales*, including metallo-β-lactamase-producing isolates. These results strongly support further clinical development of xeruborbactam combinations.

## INTRODUCTION

Restoration of β-lactam (BL) antibiotic potency with β-lactamase inhibitors (BLIs) is a proven strategy that was introduced with marketed products in the mid-80s and continues to be an important approach in the treatment of patients with infections caused by many multi-drug-resistant Gram-negative bacteria (1 – 3). Among those are carbapenem-resistant *Enterobacterales* (CRE) as well as *Enterobacterales* that are resistant to the third-generation cephalosporins. These organisms were included in the list of critical-priority pathogens posing important threats to public health that was developed by the World Health Organization in 2018 (4). BL/BLI combination products approved in 2014–2019, such as ceftolozane-tazobactam (TOL-TAZ), ceftazidime-avibactam (CAZ-AVI), meropenem-vaborbactam (MEM-VAB), and imipenem-relebactam (IMP-REL), represent a major group of antibacterial agents with useful activity against some of these pathogens (3, 5, 6). Both diazabicyclooctanes avibactam and relebactam and the boronic acid-based vaborbactam have a much broader spectrum of inhibition than older BLIs such as clavulanic acid, tazobactam, and sulbactam. However, none of these BLIs have any activity against metallo-β-lactamases (MBLs). β-Lactam resistance mediated by MBLs is of great concern as these enzymes can hydrolyze virtually all classes of β-lactams including carbapenems, cephalosporins, and penicillins (only monobactams remain resistant to hydrolysis). While bacteria producing MBLs are less prevalent compared to those producing serine β-lactamases (SBLs), the worldwide rise in their incidence during recent years is evident in both hospital and community settings (7, 8).

Two new investigational cyclic boronic acid-based BLIs, taniborbactam (TAN) (9, 10) and xeruborbactam (XER) (11, 12), are both dual SBL/MBL inhibitors. Earlier studies demonstrated that xeruborbactam inhibits multiple β-lactamases from all molecular classes including serine carbapenemases (class A KPC, class D OXA-48, and OXA-23) and metallo-β-lactamases (13, 14). Xeruborbactam has completed phase I studies (NCT04380207 and NCT04578873) administered by the intravenous or oral route (as a prodrug form). In these studies, xeruborbactam was found to be safe and well tolerated at doses of 1,000 mg/day or less and resulted in exposures that exceeded non-clinical pharmacokinetic-pharmacodynamic (PK-PD) targets (15 – 17). Taniborbactam, in combination with cefepime, has completed a phase III study of safety and efficacy in complicated urinary tract infections (NCT03840148). Taniborbactam, in combination with cefepime (18), and xeruborbactam, in combination with meropenem (19), were shown to provide broader coverage of carbapenem-resistant *Enterobacterales* compared to marketed BL/BLI combinations. One objective of this study was to determine comparative potency and spectrum of inhibition of β-lactamases by various BLIs. Another objective was to compare *in vitro* potency of XER in combination with various β-lactams to that of other BL/BLI combinations in head-to-head testing against recent isolates of CRE and *Enterobacterales* that are resistant to the third-generation cephalosporins.

## RESULTS AND DISCUSSION

### Spectrum and potency of β-lactamase inhibition by xeruborbactam in comparison to marketed BLIs and investigational BLIs in late clinical development

#### Studies with purified enzymes

Xeruborbactam and comparator BLIs *K*_*i* app_ values of inhibition (calculated with 10-minute preincubation) of several purified SBLs from structural classes A, C, and D and MBLs (structural class B) were determined and are shown in Table 1. Comparators were represented by the diazabicyclooctanes (DBOs) avibactam, relebactam, and durlobactam, all approved BLIs (durlobactam in combination with sulbactam is yet to be launched), and cyclic boronates, the approved vaborbactam and the investigational BLI taniborbactam.

**TABLE 1:** *K_i_* values (in nanomolar) of β-lactamase inhibition by xeruborbactam and comparator BLIs^*a*^

Enzyme	Class	CARB	Xeruborbactam	Vaborbactam	Avibactam	Relebactam	Taniborbactam	Durlobactam
KPC-2	A	+	1.4 ± 0.2	56 ± 15	11 ± 3	40 ± 8	32 ± 0.009	1.7 ± 0.4
CTX-M-14	A	−	0.29 ± 0.06	33 ± 13	0.41 ± 0.13	10 ± 3	1.2 ± 0.5	0.37 ± 0.16
CTX-M-15	A	−	0.37 ± 0.01	30 ± 4	0.18 ± 0.08	24 ± 1	2.6 ± 0.1	0.21 ± 0.12
SHV-12	A	−	0.74 ± 0.21	21 ± 4	0.24 ± 0.07	36 ± 6	0.15 ± 0.03	0.74 ± 0.21
TEM-10	A	−	0.66 ± 0.23	140 ± 40	1.4 ± 0.4	48 ± 7	2.8 ± 0.8	0.99 ± 0.44
P99	C	−	8.5 ± 2.4	35 ± 15	10 ± 2	14 ± 2	2.4 ± 0.5	0.17 ± 0.08
PDC1	C	−	14	ND	56	78	22	ND
OXA-48	D	+	0.22 ± 0.08	1.4 ± 0.5 × 10^4^	36 ± 10	1.8 ± 0.1 × 10^3^	6.1 ± 0.7	0.72 ± 0.27
OXA-23	D	+	0.74 ± 0.28	6.6 ± 1.1 × 10^3^	1.7 ± 0.4 × 10^3^	ND	>2 × 10^4^	94 ± 32
VIM-1	B	+	32 ± 14	>1.6 × 10^5^	>1.6 × 10^5^	>1.6 × 10^5^	31 ± 3	>1.6 × 10^5^
NDM-1	B	+	7.5 ± 2.1	>1.6 × 10^5^	>1.6 × 10^5^	>1.6 × 10^5^	4.5 ± 1.3	>1.6 × 10^5^
IMP-1	B	+	240 ± 30	>1.6 × 10^5^	>1.6 × 10^5^	>1.6 × 10^5^	>2 × 10^4^	>1.6 × 10^5^
IMP-26	B	+	4.1 × 10^3^ ± 10^3^	>1.6×10^5^	>1.6×10^5^	>1.6×10^5^	>2 × 10^4^	>2 × 10^4^
L1	B	+	1.4 × 10^3^	ND	ND	ND	1.7 × 10^4^	ND

^*a*^ Nitrocefin was used as a substrate for all the enzymes except NDM-1 and IMP-1; for these two enzymes, *K_i_* was determined using imipenem. *K*_*i* app_ values are used for class A, C, and B enzymes, and *K_i_* values are used for class B enzymes. CARB, carbapenemase; ND, not determined.

Xeruborbactam and durlobactam were the most potent BLIs against KPC-2 with nearly identical *K*_*i* app_ values in the low-nanomolar range (1–2 nM). Other BLIs were 10-fold to 50-fold less potent. Xeruborbactam, avibactam, and durlobactam had the lowest, sub-nanomolar *K*_*i* app_ values against class A extended-spectrum β-lactamases (ESBLs), exemplified by CTX-M-14/CTX-M-15, SHV-12, and TEM-10; taniborbactam was almost as potent as an ESBL inhibitor (*K*_*i* app_ values in low-nanomolar range). However, relebactam (*K*_*i* app_ values in 10- to 50-nM range) and vaborbactam (*K*_*i* app_ values in 20- to 140-nM range) were the least potent BLIs.

Durlobactam was that most potent BLI against class C P-99 (the chromosomal β-lactamase from *Enterobacter cloacae*) (*K*_*i* app_ = 0.17 nM), while xeruborbactam potency (*K*_*i* app_ = 8 nM) was similar to that of taniborbactam, avibactam, and relebactam. Against class C PDC-1 (chromosomal β-lactamase from *P. aeruginosa*), *K*_*i* app_ for all tested BLIs was in a double-digit nanomolar range with xeruborbactam and taniborbactam (*K*_*i* app_ = 14–22 nM) twofold to fivefold more potent than avibactam and relebactam (*K*_*i* app_ = 56–78 nM).

Xeruborbactam and durlobactam had the lowest *K*_*i* app_ values (sub-nanomolar range) against class D carbapenemase OXA-48 from *Enterobacterales*; potency of taniborbactam and avibactam was also relatively high, with *K*_*i* app_ values ranging from 6 to 36 nM, and neither relebactam nor vaborbactam had useful activity against this enzyme. Only xeruborbactam and durlobactam had useful activity against class D OXA-23, which is the major carbapenemase from *Acinetobacter baumannii* but was also identified in *Enterobacterales* (20). Based on *K*_*i* app_ values, xeruborbactam (*K*_*i* app_ = 0.74 nM) was ~100-fold more potent than durlobactam (*K*_*i* app_ = 94 nM).

Xeruborbactam and taniborbactam were the only BLIs that inhibited clinically important MBLs that belong to B1 sub-class, VIM-1 and NDM-1; the potency of xeruborbactam and taniborbactam was similar between these two agents, and slightly better for xeruborbactam against NDM-1 (*K*_*i*_ values of 4–7 nM) than for VIM-1 (*K*_*i*_ values of ~30 nM). Only xeruborbactam but not taniborbactam inhibited IMP MBLs: IMP-1 (with *K*_*i*_ value of 0.24 µM) and IMP-26 (with *K*_*i*_ values of 4-µM range). None of the BLIs inhibited L1 from the B3 subclass of MBLs (7).

Based on the above biochemical comparison, xeruborbactam had the broadest spectrum of β-lactamase inhibition, with the most potent inhibitory activity against many purified β-lactamases in comparison to other tested BLIs.

#### Studies using isogenic strains expressing single β-lactamases

To confirm the results of biochemical experiments and to expand information on the comparative BLI inhibition profile, we employed the large, previously described (14) panel of engineered isogenic bacterial strains expressing individual β-lactamases. Diverse β-lactamases were produced in the background of the strain PAM1154, the derivative of *P. aeruginosa* PA01 that lacks major efflux pumps. The use of this strain allows detection of β-lactamase activity [as an minimal inhibitory concentration (MIC) increase] of low catalytic efficiency enzymes that rely heavily on low permeability of the outer membrane (21) and (due to the lack of efflux) ensures appropriate interpretation of the extent of inhibition in whole-cell systems. MIC values for meropenem and cefepime alone and in the presence of xeruborbactam and the comparator BLIs (all tested at a fixed concentration of 4 µg/mL for comparative purpose) were determined and are shown in Table 2.

**TABLE 2:** MICs of meropenem and cefepime alone and in combination with BLIs against the panel of engineered *P. aeruginosa* strains producing various cloned β-lactamases

Strain	Β-lactamΒ-lactamase	Class	MEM	MEM + XER	MEMP + VAB	MEM + AVI	MEM + REL	MEM + TAN	MEM + DUR	FEP	FEP + XER	FEP + VAB	FEP + AVI	FEP + REL	FEP + TAN	FEP + DUR
PAM4175	none		**0.125^*a*^**	≤0.06	≤0.06	≤0.06	≤0.06	≤0.06	≤0.06	**0.25**	0.125	0.25	0.125	0.25	0.125	≤0.06
PAM4743	CTX-M-15	A	**0.25**	≤0.06	≤0.06	≤0.06	≤0.06	≤0.06	≤0.06	**>64**	0.125	1	0.125	1	0.25	0.125
PAM4821	CTX-M-25	A	**0.5**	≤0.06	≤0.06	≤0.06	≤0.06	≤0.06	0.125	**>64**	0.125	4	0.25	1	0.25	0.125
PAM4823	CTX-M-27	A	**0.5**	≤0.06	≤0.06	≤0.06	≤0.06	≤0.06	≤0.06	**>64**	0.125	2	0.25	2	0.25	0.125
PAM4800	GES-19	A	**1**	≤0.06	0.125	≤0.06	0.125	≤0.06	≤0.06	**64**	0.125	1	0.25	0.5	0.125	≤0.06
PAM4801	GES-20	A	**8**	≤0.06	0.25	≤0.06	0.25	≤0.06	≤0.06	**2**	0.125	0.25	0.25	0.25	0.25	≤0.06
PAM4135	KPC-2	A	**32**	≤0.06	≤0.06	≤0.06	0.125	≤0.06	≤0.06	**>64**	0.125	0.25	0.25	0.25	0.25	0.125
PAM4689	KPC-3	A	**64**	≤0.06	≤0.06	≤0.06	0.125	≤0.06	≤0.06	**>64**	0.125	0.25	0.25	0.5	0.25	0.25
PAM4825	MIR-1	C	**0.5**	≤0.06	≤0.06	≤0.06	≤0.06	≤0.06	ND	**2**	≤0.06	0.125	0.125	0.125	0.125	ND
PAM4840	OXY-6–2	A	**0.25**	≤0.06	0.125	≤0.06	≤0.06	≤0.06	≤0.06	**8**	0.125	**4**	0.25	0.25	0.25	0.125
PAM4842	PER-2	A	**0.125**	≤0.06	≤0.06	≤0.06	≤0.06	≤0.06	≤0.06	**64**	0.125	0.5	0.25	0.25	0.125	≤0.06
PAM4907	PER-4	A	**0.125**	≤0.06	≤0.06	≤0.06	≤0.06	≤0.06	≤0.06	**64**	0.125	16	**32**	**32**	0.5	0.5
PAM4874	SHV-12	A	**0.25**	≤0.06	0.125	≤0.06	≤0.06	≤0.06	≤0.06	**64**	0.125	4	0.25	1	0.25	≤0.06
PAM4744	SME-2	A	**8**	≤0.06	≤0.06	≤0.06	0.25	≤0.06	0.125	**0.5**	≤0.06	0.125	0.125	0.125	0.25	0.125
PAM4878	TEM-10	A	**0.125**	≤0.06	≤0.06	≤0.06	≤0.06	≤0.06	≤0.06	**16**	≤0.06	2	0.125	0.25	0.25	≤0.06
PAM4908	VEB-1	A	**0.5**	≤0.06	≤0.06	≤0.06	≤0.06	≤0.06	≤0.06	**>64**	0.125	2	0.25	0.25	0.25	≤0.06
PAM4910	VEB-2	A	**0.25**	≤0.06	≤0.06	≤0.06	≤0.06	≤0.06	≤0.06	**>64**	0.125	4	0.25	0.5	0.5	≤0.06
PAM4186	CMY-2	C	**0.25**	≤0.06	≤0.06	≤0.06	≤0.06	≤0.06	ND	**1**	≤0.06	0.125	≤0.06	≤0.06	≤0.06	ND
PAM4745	P99	C	**1**	≤0.06	≤0.06	≤0.06	≤0.06	≤0.06	0.125	**8**	0.125	0.25	0.125	0.125	0.125	≤0.06
PAM4869	PDC-1	C	**0.5**	≤0.06	≤0.06	≤0.06	≤0.06	≤0.06	≤0.06	**4**	0.125	0.5	0.25	0.125	0.125	0.125
PAM4884	ADC-181	C	**0.125**	≤0.06	≤0.06	≤0.06	≤0.06	≤0.06	ND	**0.5**	≤0.06	0.25	≤0.06	≤0.06	0.125	ND
PAM4827	OXA-1	D	**≤0.06**	≤0.06	≤0.06	≤0.06	≤0.06	≤0.06	≤0.06	**1**	≤0.06	**1**	≤0.06	0.25	0.25	≤0.06
PAM4792	OXA-2	D	**2**	≤0.06	**2**	0.5	0.5	≤0.06	0.125	**0.5**	≤0.06	**0.5**	0.125	0.125	0.125	≤0.06
PAM4846	OXA-9	D	**0.25**	≤0.06	≤0.06	≤0.06	≤0.06	≤0.06	≤0.06	**2**	0.125	0.25	0.125	**1**	0.125	0.125
PAM4217	OXA-48	D	**4**	≤0.06	**4**	≤0.06	**2**	0.125	0.125	**0.5**	0.125	**0.5**	0.125	0.25	0.125	0.125
PAM4875	OXA-23	D	**2**	≤0.06	**2**	0.5	**2**	**2**	≤0.06	**2**	≤0.06	**2**	0.25	**1**	**1**	≤0.06
PAM4876	OXA-72	D	**2**	≤0.06	**2**	0.5	**2**	**2**	≤0.06	**2**	≤0.06	**2**	0.25	**1**	**1**	≤0.06
PAM4179	NDM-1	B	**32**	2	**32**	**32**	**32**	2	**16**	**>64**	4	**>64**	**>64**	**>64**	8	**>64**
PAM4917	NDM-7	B	**32**	1	**32**	**32**	**32**	1	**16**	**>64**	2	**>64**	**>64**	**>64**	4	**>64**
PAM4795	VIM-1	B	**4**	≤0.06	**4**	**4**	**4**	≤0.06	4	**>64**	0.25	**>64**	**>64**	**64**	0.5	**64**
PAM4798	VIM-2	B	**8**	≤0.06	**8**	**8**	**4**	≤0.06	8	**8**	0.125	**8**	**8**	**8**	0.125	**4**
PAM4887	IMP-1	B	**4**	0.5	**4**	**4**	**4**	**4**	**4**	**64**	1	**64**	**64**	**64**	**32**	**32**
PAM4888	IMP-4	B	**4**	0.5	**4**	**2**	**4**	**2**	**2**	**32**	1	**32**	**32**	**32**	**32**	8
PAM4196	IMP-13	B	**1**	0.5	**1**	**1**	**1**	**1**	ND	**16**	1	**16**	**16**	**16**	**16**	ND
PAM4198	IMP-15	B	**1**	0.125	**1**	**1**	**1**	**1**	ND	**16**	0.5	**16**	**16**	**16**	**16**	ND
PAM4890	IMP-19	B	**1**	0.25	**1**	**1**	**1**	**1**	**0.5**	**64**	2	**32**	**32**	**32**	**32**	16
PAM4889	IMP-26	B	**8**	2	**8**	**8**	**8**	**8**	**4**	**64**	8	**64**	**64**	**64**	**64**	**32**
PAM4883	GIM-1	B	**32**	0.5	**16**	**32**	**16**	0.5	ND	**8**	0.25	**8**	**8**	**8**	0.125	ND
PAM4885	SPM-1	B	**32**	**16**	**32**	**32**	**32**	0.5	**16**	**>64**	**64**	**>64**	**>64**	**>64**	2	**64**
PAM4879	CcrA	B	**2**	≤0.06	**2**	**2**	**2**	**1**	ND	**16**	0.125	**16**	**16**	**16**	**8**	ND
PAM4880	L1	B	**32**	**16**	**32**	**32**	**32**	**32**	**16**	**4**	**1**	**4**	**4**	**4**	**4**	**1**

^*a*^ MICs for antibiotics alone and MICs of antibiotics with of BLIs that less than 4-fold lower than that of antibiotics alone are in boldfaced values.

The results of microbiological experiments were consistent with the biochemical studies. Against most strains producing class A extended-spectrum β-lactamases, all BLIs significantly increased the potency of cefepime though vaborbactam, and relebactam generally showed less potentiation compared to other BLIs, as was expected from their higher *K*_*i*_s. Lower potentiating activity of vaborbactam against the strain PAM4840 producing OXY-6-2, and vaborbactam and the DBOs avibactam and relebactam (but not the DBO durlobactam) against the PER-4-producing strain PAM4907 was noted. The highest extent of potentiation of cefepime against this group of enzymes was observed for xeruborbactam and durlobactam followed by taniborbactam. All BLIs showed a similar high extent of potentiation of both meropenem and cefepime against class A carbapenemases (KPC-2/KPC-3, SME-2, and GES-20) and cefepime against plasmidic (CMY-2 and MIR-1) and chromosomal (P99, PDC-1, and ADC-181) β-lactamases from class C.

Variable inhibitory activity of BLIs was observed against the strains producing oxacillinases, including those with carbapenemase activity, from class D. Vaborbactam demonstrated antibiotic potentiating activity (using cefepime as a reporter antibiotic) only against the OXA-9-producing strain and relebactam only against OXA-1- and OXA-2-producing strains. Taniborbactam enhanced the potency of antibiotics against the strains producing oxacillinases OXA-1/OXA-2/OXA-9 and OXA-48 (a carbapenemase) but not against carbapenemases OXA-23 and OXA-72 (the most prevalent carbapenemases in *A. baumannii*). Xeruborbactam, durlobactam, and avibactam could enhance the potency of antibiotics against all the tested class D-producing strains including those producing carbapenemases (OXA-48 and OXA-23/OXA-72), with xeruborbactam and durlobactam demonstrating the highest extent of potentiation.

Xeruborbactam and taniborbactam were the only BLIs that reduced MIC values of meropenem and cefepime against the strains producing various metallo-β-lactamases. However, as was already observed in biochemical experiments, these BLIs differed in spectrum of MBL inhibition. This was apparent using clones producing both representatives of the most prevalent MBL subfamilies, NDM, VIM, and IMP, and less prevalent GIM, SPM, and CcrA (7). While both xeruborbactam and taniborbactam showed similar extent of antibiotic potentiation against the strains producing representative NDM (NDM-1 and NDM-7) and VIM (VIM-1 and VIM-2) variants, as well as GIM-1, only xeruborbactam was able to reduce meropenem and cefepime MICs against any of the tested strains that produced MBLs from the IMP subfamily: IMP-1, IMP-4, IMP-13, IMP-15, IMP-19, and IMP-26. In addition, only xeruborbactam enhanced antibiotic potency against the strain that produced CcrA (also known as CfiA), the endogenous MBL from *Bacteroides fragilis* (22). Analysis of VIM-2 co-crystallized with taniborbactam indicated the presence of a pocket with a negative electrostatic potential, formed by E146 and D213 (conserved in various VIM variants), stabilizing the positively charged inhibitor side chain (9). This pocket is also present in most of the NDM subfamily variants and formed by E152 and D223. Interestingly, the recent study demonstrated that NDM-9 that differs from NDM-1 by a single amino acid substitution E152K (decreases electrostatic negative potential) was resistant to inhibition to by taniborbactam (23). In our own follow-up study (24), we demonstrated that the IC_50_ values of xeruborbactam inhibition of imipenem hydrolysis in cell lysates prepared from either NDM-1- or NDM-9-producing strains were essentially the same: 0.77 ± 0.12 µM and 1.2 ± 0.1 µM, respectively. This result is consistent with the analysis of co-crystals of xeruborbactam with either NDM-1 or VIM-1 that indicated that xeruborbactam does not rely on interactions with the negatively charged amino acids (important for taniborbactam binding) for binding and inhibition (11). In the same study, we showed that xeruborbactam but not taniborbactam enhanced the potency of antibiotics against NDM-9-producing clinical isolates, providing strong evidence that xeruborbactam inhibits NDM-9 that is resistant to inhibition by taniborbactam. Of note, in the case of CcrA (CfiA), in place of a negatively charged glutamate at position 131 (the expected point of interaction with taniborbactam side chain amine), there is a positively charged lysine which might contribute for resistance to inhibition by taniborbactam to this specific MBL enzyme. In IMP-1, the helix that contains a negatively charged aspartate which could interact with an amine of the inhibitor is straightened and shifts away from the ligand-binding site (bulky L124 seems to drive that shift). Together with shortened D (rather than E) side chain and several neighboring substitutions to lysines, this precludes electrostatically favorable close interaction with taniborbactam amine, providing a possible rationale for the lack of inhibition of IMP by taniborbactam.

Xeruborbactam and taniborbactam were also different in their ability to enhance meropenem and cefepime potency against the strain PAM4885 that produced SPM-1, the MBL that was discovered in 2002 in *P. aeruginosa* in Brazil (25) and until now is restricted to *P. aeruginosa* isolates from this specific region (26). In the case of SPM-1, it was taniborbactam but not xeruborbactam that enhanced antibiotic potency against PAM4885. The mechanism of this differential inhibition is under investigation.

Consistent with the biochemical results, neither xeruborbactam nor taniborbactam potentiated activity of antibiotics against the strain PAM4880 that produced L1, the chromosomally encoded MBL from the B3 subclass (7) from *Stenotrophomonas maltophilia*.

The above microbiological experiments expanded the list of β-lactamases evaluated for the comparative inhibition by different BLIs. They confirmed that xeruborbactam had the broadest spectrum of β-lactamase inhibition compared to other BLIs. They also identified several metallo-β-lactamases, e.g., NDM-9, various IMP variants, CcrA, and SPM-1, that were differentially inhibited by the two boronic acid dual SBL/MBL inhibitors, xeruborbactam and taniborbactam.

### *In vitro* potency of meropenem-xeruborbactam and comparator BL/BLI combinations against reference strains of *Enterobacterales*

The *in vitro* potency of meropenem-xeruborbactam (MEM-XER) (fixed 4 and 8 µg/mL) and comparator marketed and investigational BL/BLI combinations was determined against several publicly available strains, including the reference strains approved by the and Laboratory Standards Institute (CLSI), for quality control (QC). MEM-XER, with XER fixed 8 µg/mL, reflects the testing method recently approved by CLSI (see Material and Methods for rationale for selecting the *in vitro* testing concentration of xeruborbactam). Other BL/BLI combinations were tested at their recommended BLI concentrations. MIC values for the partner antibiotics and BLIs alone were also determined (Table 2; Table S1). All investigational and marketed BLIs enhanced the potency of antibiotics according to their spectrum of inhibition. MIC values against CLSI-approved QC strains were within acceptable ranges for drugs and combinations tested (Table 3).

**TABLE 3:** *In vitro* activity (MIC, µg/mL) of β-lactams alone, BLIs alone, and β-lactam/BLI combinations against reference β-lactamβ-lactamase-producing bacterial strains^*a*^

Strains		*Escherichia coli* NCTC 13353 (ECM7514)	*Klebsiella pneumoniae* ATCC 700603 (KPM2881)	*K. pneumoniae* ATCC BAA-1705 (KPM1123)	*K. pneumoniae* ATCC BAA-2814 (KP1074)	*P. aeruginosa* BAA-3197 (PA5257)	*E. coli* CDC 0069 (EC1106)	*K. pneumoniae* CDC 0135 (KP1291)
Β-lactamΒ-lactamases		CTX-M-15	SHV-18	KPC-2	KPC-3	KPC-2	NDM-1	VIM-1
MIC (µg/mL)	MEM	≤0.03	≤0.03	**32**	>64	>64	32	8
	MEM + XER (at 4 µg/mL)	≤0.03	≤0.03	≤0.03	0.06	2	≤0.06	≤0.06
	MEM + XER (at 8 µg/mL)	≤0.03	≤0.03	≤0.03	**≤0.03**	**2**	≤0.06	≤0.06
	MEM + VAB (at 8 µg/mL)	≤0.03	≤0.03	**≤0.03**	**0.25**	32	32	8
	FEP	**>64**	1	32	>64	>64	64	>64
	FEP + TAN (at 4 µg/mL)	**0.125**	**0.25**	**0.125**	0.5	8	0.5	1
	CAZ	64	**16**	64	>64	>64	>64	>64
	CAZ + AVI (at 4 µg/mL)	0.25	**0.5**	0.125	1	8	>64	>64
	IMP	0.125	**0.06**	8	**64**	>64	16	8
	IMP + REL (at 4 µg/mL)	0.125	**0.06**	0.125	**0.125**	4	16	8
	TOL	>64	8	64	>64	32	>64	>64
	TOL + TAZ (at 4 µg/mL)	1	**1**	64	>64	16	>64	>64
	XER	8	16	16	32	>64	16	16
	TAN	>64	>64	>64	>64	>64	>64	>64
	AVI	16	32	16	64	>64	8	>64
	REL	>64	>64	>64	>64	>64	>64	>64
	VAB	>64	>64	>64	>64	>64	>64	>64

^*a*^ AVI, avibactam; CAZ, avibactam; FEP, cefepime; IMP, imipenem; MEM, meropenem; REL, relebactam; TAN, taniborbactam; TAZ, tazobactam; TOL, ceftolozane; XER, xeruborbactam. All inhibitors were tested in combination with antibiotics at specific fixed concentrations (shown in brackets). MIC values in bold are for the strains that are CLSI-approved quality control strains for specific combinations. All these values are within acceptable QC ranges (27).

Xeruborbactam at 4 or 8 µg/mL significantly increased meropenem potency against meropenem-resistant KPC-producing strains of *Klebsiella pneumoniae* (ATCC BAA-1705 and BAA ATCC-2814), NDM-1-producing strain of *Escherichia coli* (CDC 0069), and VIM-1-producing strain of *K. pneumoniae* (CDC 0135). Taniborbactam enhanced the potency of cefepime against ESBL-, KPC-, and MBL-producing strains of *Enterobacterales*. As reported previously, direct inhibition of growth in some strains in the panel was observed for xeruborbactam (due to inhibition of multiple penicillin-binding proteins [PBPs]) (28) and avibactam (due to inhibition of PBP2) (29), but not for vaborbactam, taniborbactam, or relebactam.

### *In vitro* potency of meropenem-xeruborbactam in comparison to other BL/BLI combinations against carbapenem-resistant *Enterobacterales*

#### Surveillance isolates

The *in vitro* potency of meropenem alone or with xeruborbactam (fixed 4 or 8 µg/mL) was compared to that of cefepime-taniborbactam (FEP-TAN), ceftazidime-avibactam, meropenem-vaborbactam, and imipenem-relebactam against 1,027 CRE isolates collected as a part of the SENTRY global surveillance program in 2018–2022 (see Tables S2 and S3 for a detailed description of surveillance isolates).

Based on MIC_90_, xeruborbactam combined with meropenem at 8 µg/mL (MEM-XER) was the most potent agent vs all groups of CRE, including KPC- and OXA-48-producing isolates, carbapenemase-negative CRE, and MBL-producing isolates (Table 4). For the MBL-negative strains (*N* = 715), MEM-XER MIC_90_ strains were 0.125 µg/mL, with MIC_90_ values ranging from 2 to 32 µg/mL for other BL/BLI combinations. Against KPC-producing strains (*N* = 369), MEM-XER (MIC_90_ = 0.06 µg/mL) was 8-fold more potent than imipenem-relebactam (MIC_90_ = 0.5 µg/mL), 32-fold more potent than MEM-VAB and FEP-TAN (MIC_90_ = 2 µg/mL), and 64-fold more potent than CAZ-AVI (MIC_90_ = 4 µg/mL).

**TABLE 4:** *In vitro* activity of meropenem alone and combined with xeruborbactam at 4 and 8 µg/mL and comparator BLI combination agents against surveillance isolates of carbapenem-resistant strains of *Enterobacterales* according to carbapenemase production^*a*^

	MEM	MEM + XER at 4 µg/mL	MEM + XER at 8 µg/mL	FEP	FEP + TAN at 4 µg/mL	CAZ + AVI at 4 µg/mL	MEM + VAB at 8 µg/mL	IMP + REL at 4 µg/mL
All	*N* = 1027						*N* = 969	*N* = 507
MIC_50_	32	0.06	≤0.03	>32	1	1	4	1
MIC_90_	>32	0.5	0.25	>32	8	>32	>32	32
% Inhibited^*b*^	30.20	98.30	99.60	10.80	92.80	69.60	57	51.30
No MBL	*N* = 715						*N* = 677	*N* = 339
MIC_50_	16	0.06	≤0.03	>32	0.5	1	1	0.25
MIC_90_	>32	0.25	0.125	>32	4	2	32	4
% Inhibited	39.40	99.40	100.00	15.50	97.60	99.30	79.9	76.70
KPC	*N* = 369						*N* = 347	*N* = 193
MIC_50_	32	≤0.03	≤0.03	>32	0.25	1	≤0.03	0.125
MIC_90_	>32	0.125	0.06	>32	2	4	2	0.5
% Inhibited	29.50	98.90	100.00	11.70	99.50	99.20	98.6	97.40
OXA-48	*N* = 247						*N* = 236	*N* = 96
MIC_50_	32	0.06	≤0.03	>32	1	1	16	2
MIC_90_	>32	0.125	0.125	>32	4	2	>32	8
% Inhibited	37.20	100.00	100.00	21.50	97.60	100.00	41.9	27.10
Non-CP CRE	*N* = 99						*N* = 94	*N* = 50
MIC_50_	8	0.25	0.125	>32	2	1	1	0.25
MIC_90_	16	1	0.5	>32	8	4	4	1
% Inhibited	81.80	100.00	100.00	15.20	90.90	98.00	98.9	92
MBL	*N* = 310							*N* = 168
MIC_50_	>32	0.06	≤0.03	>32	1	>32	>32	32
MIC_90_	>32	4	1	>32	16	>32	>32	>64
% Inhibited	9.00	95.80	98.70	0.00	81.70	1.60	9.6	0
NDM	*N* = 287							*N* = 157
MIC_50_	>32	0.06	≤0.03	>32	2	>32	>32	32
MIC_90_	>32	4	1	>32	32	>32	>32	>64
% Inhibited	6.30	95.80	99.00	0.00	81.20	1.00	6.6	0
VIM	*N* = 20							*N* = 11
MIC_50_	16	≤0.03	≤0.03	32	0.5	>32	16	16
MIC_90_	>32	0.5	0.06	>32	4	>32	32	64
% Inhibited	35.00	100.00	100.00	0.00	100.00	0.00	45	0

^*a*^ AVI, avibactam; CAZ, ceftazidime; IMP, imipenem; FDA, Food and Drug Administration; FEP, cefepime; MEM, meropenem; REL, relebactam; TAN, taniborbactam; VAB, vaborbactam; XER, xeruborbactam. MIC_50_ and MIC_90_ for xeruborbactam alone were 16–32 µg/mL and 32–>32 µg/mL, respectively, for all the subsets of isolates.

^*b*^ % inhibited at the following concentrations: meropenem, ≤8 µg/mL; meropenem/xeruborbactam, ≤8/4 and ≤8/8 µg/mL; cefepime, ≤8 µg/mL (FDA susceptible breakpoint); cefepime/taniborbactam, 8/4; ceftazidime-avibactam, ≤8/4 µg/mL (FDA susceptible breakpoint); imipenem/relebactam, ≤1/4 µg/mL (FDA susceptible breakpoint); meropenem/vaborbactam, ≤4/8 (≤8/8) µg/mL (European Medicines Agency susceptible breakpoint).

Against the OXA-48-producing isolates (*N* = 248), MEM-XER (MIC_90_ = 0.125 µg/mL) was 16-fold to 32-fold more potent than CAZ-AVI (MIC_90_ = 2 µg/mL) and FEP-TAN (MIC_90_ = 4 µg/mL). As might be expected, based on the hydrolytic liability of the partner antibiotic and the BLI inhibition spectrum, neither MEM-VAB nor IMI-REL had any useful activity against OXA-48-producing strains (MIC_90_ ranging from 8 to >32 µg/mL).

Against carbapenemase-negative CRE (non-CP CRE) (*N* = 99), MEM-XER (MIC_90_ = 0.5 µg/mL) was 2-fold more potent than IMI-REL (MIC_90_ = 1 µg/mL), 8-fold more potent than MER-VAB and CAZ-AVI (MIC_90_ = 4 µg/mL), and 16-fold more potent than FEP-TAN (MIC_90_ = 8 µg/mL).

Consistent with the BLI spectrum of inhibition, only xeruborbactam and taniborbactam combinations had activity against MBL-producing isolates (*N* = 310); based on MIC_90_, MEM-XER (MIC_90_ = 1 µg/mL) was 16-fold more potent than FEP-TAN (MIC_90_ = 16 µg/mL). Against the subset of 287 NDM-producing strains, MIC_90_ of MEM-XER was 1 µg/mL as compared to 32 µg/mL for FEP-TAN; MEM-XER MIC_90_ against 20 VIM-producing isolates was 0.06 µg/mL vs 4 µg/mL for FEP-TAN. Since the inhibitory activity of xeruborbactam and taniborbactam is similar for both NDM and VIM-like enzymes, the lower MIC_90_ observed for MEM-XER compared to FEP-TAN might in part be driven by the lower MICs observed for meropenem alone than cefepime against some isolates. More studies were specifically designed to gain an additional insight into reasons for differences in potency between MER-XER and FEP-TAN (see below). The data for FEP-TAN are in close agreement with the recently reported results from the Global Evaluation of Antimicrobial Resistance via Surveillance program that involved Gram-negative bacilli from 2018 to 2020 (18). Of note, meropenem, in combination with xeruborbactam at 4 µg/mL, was also the most potent combination tested against all groups of CRE (Table 4).

As susceptibility breakpoints for MEM-XER and FEP-TAN are not yet established, we could not determine the actual coverage of CRE afforded by these combinations. For comparative purpose only, we used provisional susceptibility breakpoints of ≤8 µg/mL based on PK-PD breakpoints for the high-dose meropenem or cefepime alone (2 g every 8 hours as 3-hour infusion). MEM-XER (as well as meropenem with xeruborbactam at 4 µg/mL), FEP-TAN, and CAZ-AVI covered >95% of MBL-negative CRE, including KPC-, OXA-48-like- and non-CP-CRE-producing isolates. MER-VAB and IMI-REL covered >95% of KPC-producing isolates and non-CP CRE but, as expected, had a low coverage of OXA-48-like-producing isolates. MEM-XER covered >95% of MBL-positive isolates, most of them being NDM producers (287 out of 310) vs ~82% coverage for FEP-TAN (81.2% and 100% coverage for NDM- and VIM-producing isolates, respectively). Hence, MEM-XER (at provisional breakpoint) had the highest coverage of CRE compared to other BL/BLI combinations.

Due to weak direct antibacterial activity of xeruborbactam (MIC_50_ and MIC_90_ for xeruborbactam alone were 16–32 µg/mL and 32–>32 µg/mL, respectively, for all the subsets of isolates), mediated by the inhibition of several essential PBPs (28), some isolates of CRE (13.3% of isolates from the surveillance panel, Fig. S1) had xeruborbactam MIC of ≤8 µg/mL. For these isolates, the meropenem MICs in the presence of xeruborbactam at 8 µg/mL could not be determined precisely; MICs were reported as the lowest meropenem concentration (≤0.03 µg/mL) of a concentration range used for MIC determination (0.03–32 µg/mL). For isolates of CRE with xeruborbactam MICs of ≤8 µg/mL, we looked at the distribution of meropenem MICs determined using xeruborbactam at 4 µg/mL, a concentration which is not associated with significant growth inhibition for >99% of isolates. Ninety-five percent of isolates were inhibited at meropenem MIC of ≤0.06 µg/mL (with xeruborbactam 4 µg/mL; Fig. S1); it is expected that at least the same (or even higher) proportion of CRE isolates with xeruborbactam MIC of ≤8 µg/mL will be inhibited by meropenem at ≤0.06 µg/mL with xeruborbactam tested at 8 µg/mL. We also determined the *in vitro* potency of MEM-XER (MIC_50_/MIC_90_) for the 87% of CRE isolates from the surveillance panel that were not inhibited by xeruborbactam alone at ≤8 µg/mL; MEM-XER MIC_50_/MIC_90_ for all the subsets of CRE remained essentially unchanged (Table S4). Based on these data, we conclude that a direct antibacterial effect of xeruborbactam alone does not overestimate *in vitro* potency of MEM-XER (meropenem in combination with xeruborbactam at 8 µg/mL).

#### Challenge isolates with various defects in OmpK36/OmpC porins

Our early studies demonstrated that decreased function of the major porin OmpK36/OmpC was the major factor associated with reduced susceptibility to meropenem-xeruborbactam (19, 30). Hence, we sought to compare *in vitro* potency of meropenem-xeruborbactam to other BL/BLI combinations against CRE (*N* = 199) that had various defects in OmpK36. OmpK36 was considered either partially functional or non-functional if a duplication of two amino acids, Gly134Asp135 (GD repeat) located within the L3 internal loop resulting in the constriction of the channel (31) or frameshift, nonsense mutations, large insertions, or reduced expression of the *ompK36* gene were identified, respectively. MEM-XER potency (represented by the MIC_90_ value) against subsets of isolates with either partially functional or non-functional OmpK36/OmpC was the same, 2 µg/mL (Table 5). As expected, MEM-XER potency was lower against the challenge panel of strains with these mutations as compared to the surveillance panel that reflects current epidemiological trends (MIC_90_ = 0.25 µg/mL). For MBL-negative strains with these mutations, the MIC_90_ increased fourfold to 0.5 µg/mL; an eightfold increase in MIC_90_ was observed against MBL-positive strains (1–8 µg/mL). MEM-XER remained the most potent combination compared to the others against all subsets of CRE based on MIC_50_/MIC_90_ and covered the highest percentage of isolates based on provisional breakpoint of ≤8 µg/mL (Table 5). Three out of 199 isolates, with a defective OmpK36, had MEM-XER MIC values of >8 µg/mL. All three isolates produced NDM-type MBLs (two NDM-1 and one NDM-7). The mechanisms governing the increased MEM-XER MIC values in these particular NDM-producing isolates are under investigation.

**TABLE 5:** *In vitro* activity of meropenem plus xeruborbactam at 4 and 8 µg/mL and comparator BLI combination agents and comparator BLI combination agents against carbapenem-resistant strains of *Enterobacterales* with either partially functional or non-functional OmpK36 according to carbapenemase production^*a*^

	MEM + XER (4)	MEM + XER (8)	FEP + TAN (4)	CAZ + AVI (4)	MEM + VAB (8)	IMP + REL (4)
PFN^*b*^ OmpK36	*N* = 137					
MIC_50_	0.25	0.125	2	4	16	2
MIC_90_	8	2	32	>64	>64	>64
% Inhibited^*c*^	91.2	98.5	80.3	68.6	49.6	47.4
Non-functional (NF)^*b*^ OmpK36	*N* = 62					
MIC_50_	0.5	0.25	2	4	8	4
MIC_90_	16	2	8	>64	>64	>64
% Inhibited	83.9	96.8	90.3	67.7	61.3	24.2
PFN + NF MBL negative	*N* = 154					
MIC_50_	0.25	0.125	2	2	4	1
MIC_90_	4	0.5	8	16	64	8
% Inhibited	92.90	100.00	98.10	88.30	68.20	51.90
PFN + NF KPC	*N* = 118					
MIC_50_	0.25	0.125	2	4	2	1
MIC_90_	8	0.5	8	16	64	8
% Inhibited	91.50	100.00	97.50	84.70	78.80	63.60
PFN + NF OXA-48-like	*N* = 31					
MIC_50_	0.125	≤0.06	2	2	32	4
MIC_90_	0.25	0.25	8	4	64	64
% Inhibited	100.00	100.00	100.00	100.00	22.60	3.20
PFN + NF all MBLs	*N* = 45					
MIC_50_	4	1	32	>64	>64	>64
MIC_90_	64	8	>64	>64	>64	>64
% Inhibited	75.60	91.10	33.30	0.00	2.20	0
PFN + NF NDM	*N* = 29					
MIC_50_	8	2	32	>64	>64	>64
MIC_90_	64	16	>64	>64	>64	>64
% Inhibited	68.90	86.20	31.00	0.00	0.00	0.00
PFN + NF VIM	*N* = 13					
MIC_50_	2	0.25	16	>64	>64	>64
MIC_90_	8	1	32	>64	>64	>64
% Inhibited	92.30	100.00	46.20	0.00	0.00	0.00

^*a*^ AVI, avibactam; CAZ, avibactam; FEP, cefepime; IMP, imipenem; MEM, meropenem; NF, non-functional; PFN, partially functional; REL, relebactam; TAN, taniborbactam; VAB, vaborbactam; XER, xeruborbactam.

^*b*^ OmpK36 was considered PFN if it contained a duplication of two amino acids, Gly134Asp135 (GD repeat) located within the L3 internal loop, resulting in the constriction of the channel; OmK36 was considered NF if it contained frameshift, nonsense mutations and large insertions, or reduced expression due to insertions in the promoter region has been identified.

^*c*^ % Inhibited at the following concentrations: meropenem/xeruborbactam: ≤8/4 and ≤8/8 µg/mL; cefepime/taniborbactam: ≤8/4; ceftazidime-avibactam : ≤8/4 µg/mL (FDA susceptible breakpoint); imipenem/relebactam: ≤1/4 µg/mL (FDA susceptible breakpoint); meropenem/vaborbactam: ≤8/8 µg/mL (European Medicines Agency susceptible breakpoint). BLI concentrations are in brackets.

#### CRE with reduced susceptibility for cefepime-taniborbactam

**Fig 1:**
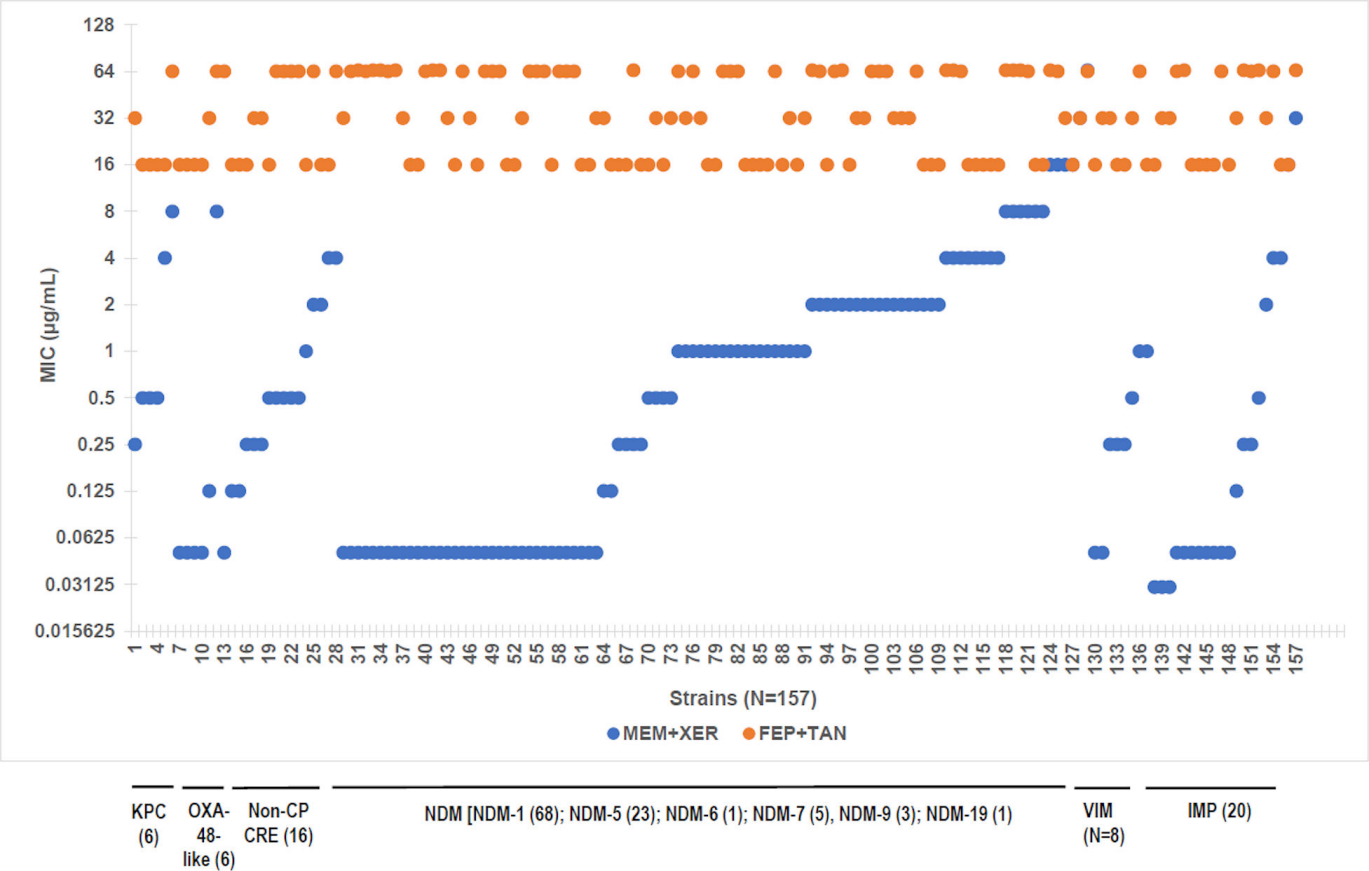
*In vitro* potency of meropenem in combination with xeruborbactam against CRE with cefepime-taniborbactam MIC values of >8 µg/mL. MEM-XER at 8 µg/mL, FEP-TAN at 4 µg/mL. FEP-TAN, cefepime-taniborbactam; MEM-XER, meropenem-xeruborbactam.

We next determined *in vitro* potency of meropenem-xeruborbactam (4 and 8 µg/mL) against the subset of 157 isolates with an increased MIC value of cefepime-taniborbactam (MIC >8 µg/mL). Such isolates were present in various subsets of CREs, including both MBL-positive (NDM, VIM, and IMP) and MBL-negative (KPC, OXA-48-like, and non-CP CRE) strains (Table 6; Fig. 1). Increased FEP-TAN MIC against IMP-producing or NDM-9-producing isolates (*N* = 20 and *N* = 3 in the current panel, respectively) can be explained by the inability of taniborbactam to inhibit these MBLs. However, most of the strains with FEP-TAN MIC values of >8 µg/mL carried β-lactamases that are susceptible to inhibition by taniborbactam. This indicates the presence of non-β-lactamase-mediated resistance. Recent studies (18, 32) implicated porin mutations, increased efflux, and PBP3 mutations (in particular insertions of four amino acids, YRIN, or YRIK) in increasing FEP-TAN MIC values.

**TABLE 6:** *In vitro* activity of meropenem alone and combined with xeruborbactam at 4 and 8 µg/mL and comparator BLI combination agents against carbapenem-resistant strains of *Enterobacterales* with cefepime-taniborbactam MIC >8 µg/mL

	MEM	MEM + XER (4)	MEM + XER (8)	FEP	FEP + TAN (4)	CAZ + AVI
	All (*N* = 156)					
MIC_50_	>64	2	0.5	64	32	>64
MIC_90_	>64	32	8	>64	>64	>64
	MBL negative (*N* = 34)					
MIC50	16	0.5	0.25	64	16	4
MIC90	>64	8	4	>64	64	32
	KPC and OXA-48 (*N* = 12)					
MIC_50_	32	0.5	0.25	64	16	2
MIC_90_	>64	16	8	>64	64	64
	Non-CP CRE (*N* = 22)					
MIC_50_	16	1	0.5	64	32	2
MIC_90_	32	8	4	>64	64	32
	All MBL (*N* = 129)					
MIC_50_	>64	4	0.5	64	32	>64
MIC_90_	>64	32	8	>64	>64	>64
	NDN (*N* = 101)					
MIC_50_	>64	4	1	64	32	>64
MIC_90_	>64	32	8	>64	>64	>64
	VIM (*N* = 8)					
MIC_50_	>64	2	0.25	>64	16	>64
	IMP (*N* = 20)					
MIC_50_	16	0.5	≤0.06	32	32	>64
MIC_90_	64	16	4	>64	>64	>64

MIC_50_/MIC_90_ for meropenem-xeruborbactam (4 µg/mL) and meropenem-xeruborbactam (8 µg/mL, MEM-XER) were 2 µg/mL/32 µg/mL and 0.5 µg/mL/4 µg/mL, respectively, vs 32 µg/mL/>64 µg/mL for FEP-TAN (Table 6). MIC_90_ values of MEM-XER against the MBL-negative subset (*N* = 28) was 4 µg/mL compared to 64 µg/mL for FEP-TAN. None of the MBL-negative isolates had MEM-XER MIC values of >8 µg/mL. Nevertheless, MEM-XER MIC_90_ values were higher compared to that for the MBL-negative surveillance (MIC_90_ = 0.125 µg/mL) (Table 4), indicating some degree of cross resistance. *OmpK36/ompC* sequences were available for ~25% of these isolates, and all of them carried various defects in these genes. This pointed to the high proportion of *ompK36/ompC* mutants among MBL-negative isolates with increased FEP-TAN MIC values. At the same time, MEM-XER MIC_90_ for MBL-negative OmpK36-defective isolates was much lower, 0.5 µg/mL. This indicates that additional mechanisms operating in the background of OmpK36 defects contribute to the increased MEM-XER values against this subset of isolates. The possible mechanisms are currently under investigation.

MIC_90_ value of MEM-XER against the subset of 129 MBL positive was 8 µg/mL compared to >64 µg/mL for FEP-TAN. MEM-XER MIC values ranged from ≤0.06 to >64 µg/mL vs 16 to >64 µg/mL ranges for FEP-TAN (Fig. 1). MEM-XER MIC values were <8 µg/mL for 121 out of 129 MBL-producing isolates (93.7%): 93 out of 101 NDM-producing, 8 out of 8 VIM-producing, and 18 out of 20 IMP-producing isolates. Out of 129 isolates, 53 had MEM-XER MIC values of ≤0.06 µg/mL, 35 of them producing NDM variants [NDM-1, NDM-5, or NDM-7 that are inhibited by taniborbactam (23)]. We have previously determined *ompK36/ompC* sequences for 19 of these isolates (19), and all of them carried functional OmpK36/OmpC. *ftsI* (encodes PBP3) sequence was available for 11 out of 19 isolates, and all 11 carried YRIN insertion. Thus, isolates that produce NDM variants that are inhibited by taniborbactam and have functional OmpK36/OmpC might have increased FEP-TAN MIC associated (at least in part) with a four-amino acid insertion in PBP3. Such isolates remain highly susceptible to MEM-XER (MIC ≤0.06 µg/mL).

MEM-XER MIC values were >8 µg/mL for 8 out of 129 isolates: 5 out of 68 NDM-1-producing strains, 1 out of 5 NDM-7-producing strains, and 2 out of 20 IMP-producing isolates (1 out of 6 IMP-4-producing strains and a single IMP-64-producing isolate). Our previous analysis indicated that these strains have various defects in OmpK36/OmpC. Identification of additional mechanisms that contribute to the increased MEM-XER MIC in these isolates is in progress. Of a particular note, none of the isolates from either the surveillance or the OmpK36 challenge panel with FEP-TAN MIC of ≤8 µg/mL had MEM-XER MIC of >8 µg/mL; in fact, 100% of these isolates were inhibited by ≤2 µg/mL of MEM-XER.

In summary, 100% of isolates with FEP-TAN MIC values of ≤8 µg/mL and >90% of isolates with increased FEP-TAN MIC values (MIC >8 µg/L are inhibited by <8 µg/mL of MEM-XER, with many of them having an MIC as low as ≤0.06 µg/mL. No isolates with MEM-XER MIC values of >8 µg/mL and having FEP-TAN MIC values of <8 µg/mL have been identified to date.

### Comparative potency of xeruborbactam or taniborbactam in combination with meropenem and cefepime against CRE

As xeruborbactam and taniborbactam were the only BLIs with inhibitory activity against both serine and metallo-β-lactamases, we performed additional studies to gain insights into differences between these BLIs. We compared potency of xeruborbactam (fixed 4 and 8 µg/mL) and taniborbactam (4 µg/mL) combined with meropenem and cefepime against CRE isolates. In a panel of OmpK36-proficient CRE isolates that were MBL negative, the MIC_90_ xeruborbactam (fixed 4 µg/mL) in combination with either meropenem or cefepime was 4-fold to 16-fold more potent than the respective taniborbactam (fixed 4 µg/mL) combinations. The difference was amplified 8-fold to 32-fold when the fixed xeruborbactam testing concentration was 8 µg/mL. Against OmpK36-proficient MBL-positive isolates (represented by NDM- and VIM-producing strains), xeruborbactam (tested at a fixed 4 µg/mL) combinations were fourfold to eightfold more potent than taniborbactam (fixed 4 µg/mL) combinations and 16-fold more potent with xeruborbactam tested at a fixed 8 µg/mL (Table 7). OmpK36 deficiency reduced *in vitro* potency of all combinations; however, xeruborbactam, in combination with either meropenem or cefepime (fixed 4 µg/mL), was more potent than taniborbactam combinations (fixed 4 µg/mL) against both MBL-negative and MBL-positive isolates. For MBL-positive strains, based on MIC_50_, xeruborbactam combinations were 4-fold to 8-fold more potent than taniborbactam combinations and 16-fold to 32-fold more potent with xeruborbactam tested at 8 µg/mL (Table 7).

**TABLE 7:** *In vitro* activity of meropenem and cefepime combined with xeruborbactam or taniborbactam against carbapenem-resistant strains of *Enterobacterales* with a functional or defective OmpK36 according to carbapenemase production^*a*^

	MEM + XER (4)	MEM + XER (8)	MEM + TAN (4)	FEP + XER (4)	FEP + XER (8)	FEP + TAN (4)	MEM + XER (4)	MEM + XER (8)	MEM + TAN (4)	FEP + XER (4)	FEP + XER (8)	FEP + TAN (4)
	OmpK36 **^*b*^**						OmpK36 defective					
All	*N* = 173						*N* = 199					
MIC_50_	≤0.06	≤0.06	0.25	0.125	≤0.06	1	0.25	0.125	4	1	0.25	2
MIC_90_	0.25	≤0.06	4	4	0.5	16	16	2	32	8	4	32
No MBLs	*N* = 46						*N* = 154					
MIC_50_	≤0.06	≤0.06	≤0.06	0.125	≤0.06	0.25	0.25	0.125	1	0.5	0.25	2
MIC_90_	0.125	≤0.06	0.5	0.25	0.125	2	4	0.5	16	4	1	8
KPC	*N* = 36						*N* = 118					
MIC_50_	≤0.06	≤0.06	≤0.06	≤0.06	≤0.06	0.25	0.25	0.125	1	0.5	0.25	2
MIC_90_	≤0.06	≤0.06	0.125	0.25	0.125	1	8	0.5	16	4	1	8
OXA-48-like	*N* = 10						*N* = 31					
MIC_50_	≤0.06	≤0.06	0.25	0.125	≤0.06	0.25	0.125	≤0.06	4	0.5	0.25	2
MIC_90_	0.125	≤0.06	4	0.5	0.125	4	0.25	0.25	8	2	0.5	8
All MBL	*N* = 117						*N* = 45					
MIC_50_	≤0.06	≤0.06	0.5	0.25	≤0.06	1	4	1	16	4	1	32
MIC_90_	0.25	0.125	2	4	1	16	64	8	>64	64	16	>64
NDM	*N* = 104						*N* = 29					
MIC_50_	0.05	0.05	0.5	0.25	0.05	2	8	2	32	8	2	32
MIC_90_	0.25	0.125	4	4	1	16	64	16	>64	>64	16	>64
VIM	*N* = 13						*N* = 13					
MIC_50_	≤0.06	≤0.06	≤0.06	0.125	≤0.06	0.5	2	0.25	8	2	0.125	16
MIC_90_	≤0.06	≤0.06	0.125	0.5	≤0.06	1	8	1	32	8	1	32

^*a*^ FEP, cefepime; MEM, meropenem; TAN, taniborbactam; XER, xeruborbactam.

^*b*^ OmpK36 was considered defective if frameshift, nonsense mutations, large insertions, a duplication of two amino acids, Gly134Asp135 (GD repeat) located within the L3 internal loop resulting in the constriction of the channel, or reduced expression due to insertions in the promoter region have been identified.

Since xeruborbactam is generally a more potent inhibitor of various serine β-lactamases, including KPC and OXA-48, the higher *in vitro* potency of xeruborbactam combinations compared to taniborbactam combinations against MBL-negative CRE clinical isolates was expected.

At the same time, our studies with purified enzymes demonstrated that both BLIs had nearly identical *K_i_* s against purified NDM-1 and VIM-1 (Table 1); hence, the higher potency of xeruborbactam combinations against NDM- and VIM-producing isolates could not be explained simply by biochemical results. To explore this, we compared the relative uptake of both BLIs across the outer membrane of *K. pneumoniae*. The relative uptake of BLIs was assessed based on the ratio between IC_50_ of inhibition of a β-lactamase activity in whole cells vs IC_50_ of inhibition of a β-lactamase inhibition in cell lysates; the higher the ratio, the lower the permeability of a BLI across the outer membrane. Reporter β-lactamases were NDM-1 and VIM-1; NDM-1 was produced by cells of KP1081 that had a functional OmpK36 porin, and VIM-1 was produced by cells of KP1015 with a partially inactivated OmpK36 (GD repeat). β-Lactamase activity was monitored based on the rate of cleavage of imipenem added to whole cells or cell lysates. The results of this evaluation (Table 8) demonstrated that (i) both BLIs had similar potency of inhibition against NDM-1 and VIM-1 (judged from the IC_50_ of inhibition in cellular lysates); (ii) xeruborbactam had a slightly (1.5-fold) higher uptake across the outer membrane of cells with functional OmpK36 compared to taniborbactam (relative IC_50_ of 21.5 for xeruborbactam vs 30 for taniborbactam); and (iii) taniborbactam had a slightly (1.5-fold) higher uptake across the outer membrane of cells with defective OmpK36 compared to xeruborbactam (relative IC_50_ of 280 for xeruborbactam vs 200 for taniborbactam). Therefore, while faster uptake may contribute to the increased potency of xeruborbactam combinations vs taniborbactam combinations against MBL-positive OmpK36-proficient strains, it did not fully explain the magnitude of difference in whole-cell potency observed between BLI combinations or the results obtained against MBL-positive OmpK36-deficient strains.

**TABLE 8:** Relative uptake of xeruborbactam and taniborbactam across the outer membrane of *K. pneumoniae* cells^*c*^

				IC_50_ (µM) of β-lactamase inhibition in cell lysates		IC_50_ (µM) of β-lactamase inhibition in whole cells		IC_50_ ratio^*a*^		MIC (µg/mL)^*b*^			
**Strain**	**OmpK35**	**OmpK36**	**MBL**	**XER**	**TAN**	**XER**	**TAN**	**XER**	**TAN**	**MEM + XER**	**MEM + TAN**	**FEP + XER**	**FEP + TAN**
KP1081	TAG at aa #92	Full length	NDM-1	0.77 ± 0.12	0.24 ± 0.052	16.6 ± 1.0	7.2 ± 0.2	21.5	30	0.5	4		8
KP1015	TAA at aa #173	GD repeat	VIM-1	0.25 ± 0.02	0.19 ± 0.01	70.0 ± 13.4	39.1 ± 8.2	280	200	8	16	4	32

^*a*^ Ratio between IC_50_ in whole cells vs IC_50_ in cell lysates. The higher the ratio, the lower is the rate of uptake of a BLI across the outer membrane.

^*b*^ BLIs at 4 µg/mL.

^*c*^ FEP, cefepime; MER, meropenem; TAN, taniborbactam; XER, xerubornactam.

One feature that differentiates xeruborbactam and taniborbactam is that xeruborbactam has some intrinsic antimicrobial activity that has been attributed to inhibition of multiple essential PBPs (28). In contrast, taniborbactam has been reported to lack direct antibacterial activity at concentrations of >512 µg/mL (9). PBP inhibition might result in an additional synergy between xeruborbactam and certain partner β-lactam antibiotics; for example, a greater extent of meropenem potentiation by xeruborbactam compared to vaborbactam, which, similar to taniborbactam, lacks direct antibacterial activity at concentrations associated with a complete inhibition of the KPC β-lactamase (28). We used a panel of isogenic engineered porin/efflux mutants of *K. pneumoniae* and their derivatives producing NDM-1 to assess the effect of xeruborbactam and taniborbactam on meropenem and cefepime MIC. Xeruborbactam (fixed 4 or 8 µg/mL) reduced meropenem and cefepime MIC in the strains that lack β-lactamases twofold to eightfold (Table 9). Taniborbactam tested at the same concentrations did not have any effects on meropenem or cefepime MICs in these strains. In the case of NDM-1-producing strains, xeruborbactam (fixed 4 µg/mL) nearly completely reversed NDM-1-mediated meropenem and cefepime resistance in the wild-type strain (KPM1281) and the mutant with an increased efflux (KPM1282). Xeruborbactam (fixed 8 µg/mL) reduced meropenem and cefepime MICs below the level observed for the corresponding NDM-1-lacking strains (KPM1026a and KPM1027). Meropenem and cefepime MICs with taniborbactam (fixed 4 and 8 µg/mL) were twofold to eightfold higher than that with xeruborbactam (fixed 4 and 8 µg/mL). The difference between meropenem and cefepime MICs with xeruborbactam or taniborbactam was still twofold to fourfold or fourfold to eightfold with BLIs tested at 4 or 8 µg/mL, respectively, in the NDM-1-producing mutants lacking either OmpK36 alone (KPM3820) and both OmpK35 and OmpK36 (KPM3825).

**TABLE 9:** *In vitro* activity of meropenem or cefepime combined with xeruborbactam or taniborbactam against isogenic efflux and porin mutants of *K. pneumoniae* and their NDM-1-producing derivatives

Strain	Genotype^*a*^	Β-lactamΒ-lactamase	BLI	Meropenem MIC (µg/mL) with BLIs (µg/mL)			Cefepime MIC (µg/mL) with BLIs (µg/mL)			BLI MIC (µg/mL)
				0	4	8	0	4	8	
KPM1026a	Wild type, ATCC 43816	None	XER	0.03	0.015	0.008	0.03	0.015	0.002	16
			TAN	0.03	0.03	0.03	0.03	0.03	0.03	>64
KPM1281		NDM-1	XER	32	0.06	0.004	16	0.03	0.008	16
			TAN	32	0.125	0.06	16	0.25	0.125	>64
KPM1027	ramR*	None	XER	0.06	0.015	0.004	0.25	0.125	0.015	16
			TAN	0.06	0.06	0.06	0.25	0.25	0.25	>64
KPM1282		NDM-1	XER	64	0.125	0.03	32	0.25	0.06	16
			TAN	64	0.25	0.125	32	0.5	0.5	>64
KPM2040	mpK36_2040*	None	XER	0.125	0.06	0.03	0.125	0.06	0.015	32
			TAN	0.125	0.125	0.125	0.125	0.125	0.125	>64
KPM3820		NDM-1	XER	128	4	1	128	1	0.5	32
			TAN	128	8	4	128	4	2	>64
KPM2013	*mpK36_2040 ΔompK35*	None	XER	0.25	0.125	0.06	0.5	0.25	0.06	32
			TAN	0.25	0.25	0.25	0.5	0.5	0.5	>64
KPM3825		NDM-1	XER	256	8	2	256	2	1	32
			TAN	256	32	16	256	8	4	>64

^*a*^ *ramR*, 18-bp insertion causing frameframeshift from aa#46 (results in overexpression of the *acrAB* efflux operon and downregulation of the *ompK35* gene); ompK36_2040, the frameshift from aa#54.

Taken together, the above results are consistent with the possibility that the greater extent of β-lactam potentiation (lower potentiated MICs) observed with xeruborbactam as compared to taniborbactam in β-lactamase-producing strains (including those that are inhibited with the same potency by these BLIs) is due to an additional enhancement through direct antimicrobial activity based on PBP inhibition exerted by xeruborbactam but not taniborbactam. It appears that the potent broad-spectrum β-lactamase inhibition by xeruborbactam, combined with its intrinsic antibacterial activity, provides added potency benefit to multiple xeruborbactam/β-lactam combinations.

### Comparative *in vitro* potency of xeruborbactam in combination with multiple β-lactam antibiotics against ESBL-producing, carbapenem-resistant *Enterobacterales*

In addition to xeruborbactam-meropenem, *in vitro* potency of xeruborbactam (fixed 8 µg/mL), in combination with several other β-lactam antibiotics, cefepime, ceftolozane, ceftriaxone, aztreonam, piperacillin, and ertapenem, was determined against 521 clinical isolates of *Enterobacterales* that carried various class A, class C, and class D extended-spectrum β-lactamases and the subset of 507 carbapenem-resistant isolates (Table 10). Significant potentiation of all antibiotics by xeruborbactam was observed against each subset of isolates. Against ESBL-producing strains, the MIC_90_ values for xeruborbactam combinations with non-carbapenem antibiotics were generally lower compared to other combinations. For example, MIC_90_ values were ≤0.03 µg/mL (cefepime, ceftriaxone, and aztreonam with xeruborbactam), 0.125 µg/mL (ceftolozane with xeruborbactam), and 0.5 µg/mL (piperacillin with xeruborbactam); for comparison, MIC_90_ for cefepime-taniborbactam and ceftazidime-avibactam were 0.25 and 1.0 µg/mL, respectively. MIC_90_ for all the tested xeruborbactam combinations and for cefepime-taniborbactam were in a susceptible range for antibiotics alone.

**TABLE 10:** *In vitro* activity of xeruborbactam combinations with multiple antibiotics and comparator BLI combination agents against ESBL-producing and carbapenem-resistant *Enterobacterales*

	MEM	MEM + XER	FEP	FEP + XER	FEP+ TAN	C/T	C/T + XER	ATM	ATM + XER	TZP	TZP + XER	CRO	CRO + XER	ETP	ETP + XER	MVB	I-R	CZA
All (*N* = 1028)																		
MIC_50_	0.5	≤0.03	64	≤0.03	0.125	8	0.06	64	≤0.03	128	0.125	>32	≤0.03	4	0.02	≤0.03	0.125	0.5
MIC_90_	>64	0.06	>64	0.25	2	>64	4	>64	0.125	>128	2	>32	0.5	>32	0.25	64	16	>32
ESBL (*N* = 521)																		
MIC_50_	≤0.03	≤0.03	16	≤0.03	0.06	0.5	≤0.03	32	≤0.03	4	0.05	>32	≤0.03	0.06	≤0.03	≤0.03	0.125	0.25
MIC_90_	0.06	≤0.03	>64	≤0.03	0.25	8	0.125	>64	≤0.03	128	0.5	>32	≤0.03	0.5	≤0.03	≤0.03	0.25	1
CRE (*N* = 507)																		
MIC_50_	0.5	≤0.03	>64	≤0.03	0.5	>64	0.25	>64	≤0.03	>128	1	>32	0.125	>32	≤0.03	2	1	2
MIC_90_	>64	0.06	>64	0.5	8	>64	16	>64	0.25	>128	4	>32	1	>32	0.5	>64	32	>32
KPC (*N* = 193)																		
MIC_50_	16	≤0.03	>64	≤0.03	0.25	64	0.125	>64	≤0.03	>128	0.5	>32	≤0.03	>32	≤0.03	≤0.03	0.125	1
MIC_90_	>64	0.06	>64	0.25	1	>64	0.5	>64	0.125	>128	2	>32	0.25	>32	0.125	0.5	0.5	2
OXA-48-like (*N* = 96)																		
MIC_50_	16	≤0.03	>64	0.06	1	64	0.25	>64	0.06	>128	1	>32	0.125	>32	0.06	16	2	1
MIC_90_	64	0.125	>64	1	4	>64	1	>64	0.25	>128	16	>32	0.5	>32	0.5	32	8	2
Non-CP CRE (*N* = 50)																		
MIC_50_	4	0.06	>64	0.125	2	64	0.25	>64	0.125	>128	2	>32	0.125	>32	0.25	1	0.25	1
MIC_90_	16	0.5	>64	0.5	8	>64	0.5	>64	0.5	>128	8	>32	0.5	>32	1	4	1	2
MBL (*N* = 168)																		
MIC_50_	64	≤0.03	>64	≤0.03	1	>64	8	>64	≤0.03	>128	0.5	>32	0.25	>32	≤0.03	64	32	>32
MIC_90_	>64	1	>64	0.5	16	>64	64	>64	0.125	>128	4	>32	4	>32	1	>64	>64	>32

^*a*^ MEM, meropenem; FEP, cefepime; CZA, ceftazidime-avibactam; I-R, imipenem-relebactam; C/T, ceftolozane-tazobactam; PIP, piperacillin; TZP, piperacillin-tazobactam; CRO, ceftriaxone; ETP ertapenem; XER, xeruborbactam; TAN, taniborbactam. Xeruborbactam and vaborbactam were tested at a fixed concentration of 8 mg/L, all other BLIs were at 4 mg/L.

Against KPC-producing strains, based on MIC_90_, xeruborbactam combined with meropenem (MIC_90_ = 0.06 µg/mL), ertapenem (MIC_90_ = 0.125 µg/mL), cefepime (MIC_90_ = 0.25 µg/mL), ceftriaxone (MIC_90_ = 0.25 µg/mL), and aztreonam (MIC_90_ = 0.125 µg/L) was more potent than any of comparator combinations (MIC_90_ ranging from 0.5 to 2.0 µg/mL, Table 10). Against this subset of isolates, MIC_90_ values for all xeruborbactam combinations and cefepime-taniborbactam were at or below susceptibility breakpoints for antibiotics alone. MIC_90_ values for the approved BL/BLI combinations were also below approved susceptibility breakpoints. Against OXA-48-producing CRE, xeruborbactam combinations with meropenem (MIC_90_ = 0.125 µg/mL), ertapenem (MIC_90_ = 0.5 µg/L), cefepime (MIC_90_ = 1 µg/mL), ceftolozane (MIC_90_ = 1 µg/mL), ceftriaxone (MIC_90_ = 0.5 µg/mL), and aztreonam (MIC_90_ = 0.25 µg/mL) were more potent than cefepime-taniborbactam MIC_90_ = 4 µg/mL) or ceftazidime-avibactam (MIC_90_ = 2 µg/mL). All these MIC_90_ values were below susceptibility breakpoints for antibiotics alone. MIC_90_ values of xeruborbatam in combination with meropenem, cefepime, ceftolozane-tazobactam, aztreonam, and ceftriaxone were 0.5 µg/mL, the lowest values compared to that of other BL/BLI combinations and in a susceptible range for antibiotics alone. For comparison, MIC_90_ for cefepime-taniborbactam was 8 µg/mL (susceptible dose-dependent breakpoint for cefepime alone). MIC_90_ values for all approved BL/BLI combinations were in a susceptible range for this subset of CREs. Finally, based on MIC_90_, all xeruborbactam combinations were more potent against MBL-producing isolates compared to cefepime-taniborbactam with MIC_90_ = 16 µg/mL, which falls within resistance range for cefepime alone. MIC_90_ values of xeruborbactam combined with meropenem (MIC_90_ = 1 µg/mL), cefepime (MIC_90_ = 0.5 µg/L), aztreonam (MIC_90_ = 0.125 µg/mL), and piperacillin-tazobactam (MIC_90_ = 4 µg/mL) were in a susceptible range for antibiotics alone (Table 10).

In summary, xeruborbactam significantly enhanced the activity of multiple β-lactam antibiotics against various subsets of CRE and ESBL-producing *Enterobacterales* and was able to shift >90% of isolates below susceptibility breakpoints of multiple β-lactams.

### Summary

This study provides comprehensive head-to-head comparison of the biochemical and *in vitro* microbiological activity of various marketed and investigational BLIs and BL/BLI combinations involving large panels of carbapenem-resistant *Enterobacterales*. Based on biochemical experiments with purified enzymes and microbiological experiments using cloned β-lactamases, xeruborbactam was confirmed to have the broadest spectrum and often the highest potency of inhibition of the most prevalent and clinically important β-lactamases from all molecular classes. Unlike the DBO BLIs, it is a dual inhibitor of both serine and metallo-β-lactamases. Compared to another SBL/MBL dual inhibitor taniborbactam, xeruborbactam has a broader spectrum of inhibition of both serine and metalloenzymes. With serine enzymes, the important differentiating feature of xeruborbactam is its ability to inhibit class D carbapenemases such as OXA-23 that are not inhibited by taniborbactam. With respect to MBLs, the xeruborbactam, but not taniborbactam, spectrum includes multiple representatives of the IMP subfamily as well as emerging NDM variants such as NDM-9 identified in many critically important Gram-negative pathogens including carbapenem-resistant *Enterobacterales* (33, 34).

Compared to other studied BL/BLI combinations, MER-XER (xeruborbactam at 8 µg/mL) (as well as xeruborbactam at 4 µg/mL) was the most potent agent vs CRE in both the surveillance panel of CRE and the challenge panel consisting of OmpK36 mutants. It also provided the highest coverage at the provisional breakpoint of 8 µg/mL; MEM-XER and FEP-TAN were the only BL/BLIs with *in vitro* activity against MBL producers. Based on MIC_90_, MEM-XER (MIC_90_ of 1 mg/L) was 16-fold more potent than FEP-TAN (MIC_90_ of 16 mg/L). MIC values of MEM-XER were equal or below 8 µg/mL for >90% of CRE with FEP-TAN MIC values above 8 µg/mL.

In addition to meropenem, xeruborbactam significantly increased the potency of other β-lactams against *Enterobacterales*, providing the basis for its potential use with multiple β-lactam antibiotics.

## MATERIALS AND METHODS

### β-Lactamase enzyme preparations

The majority of purified β-lactamase enzymes used in the study were either expressed and purified internally (35) or obtained from Emerald Biostructures (Bainbridge Island, WA, USA). L1 enzyme was purified as described in reference (36).

### Determination of *K_i_*_app_ values of BLIs for various β-lactamases with nitrocefin or imipenem as a substrate

Enzymes were mixed with BLIs at concentrations varying from 160 to 0.0027 µM in 50-mM Na-phosphate pH7.0, 0.1-mg/mL bovide serum albumin (BSA) (buffer A; 20-µM ZnCl_2_ was also added for all metalloenzymes) and incubated for 10 minutes at 37°C. Nitrocefin (50 µM) (10 µM for SHV-12) or 100 µM of imipenem (prewarmed at 37°C for 10 minutes) was added, and substrate cleavage profiles were recorded at 37°C at 490 nm every 10 seconds for 10 minutes or at 294 nm every 30 seconds for 1 hour for nitrocefin and imipenem, respectively. Substrate concentrations for *K_i_* determinations were selected not to exceed *K_m_* values by >2-fold to prevent the “saturation” of enzyme activity with substrate. *K_i_*_app_ values (*K_i_* for metalloenzymes) were calculated by the method of Waley (37).

### Statistical analysis

All kinetic results are presented as average ± standard deviation of minimum three replicates.

### Bacterial strains

The panel of engineered isogenic strains of *P. aeruginosa* producing 40 individual β-lactamases was used to compare the profiles of β-lactamase inhibition by xeruborbactam and comparator BLIs. The construction of recombinant β-lactamase-producing plasmids (based on a vector plasmid pUCP24, carrying a gentamicin resistance gene) was described previously (38); the complete set of primers used to amplify various genes is provided in reference (14). The test panel of Gram-negative bacteria consisted mainly of worldwide isolates of carbapenem-resistant *Enterobacterales*, collected in 2018–2020 as a part of the global SENTRY program; it was geographically diverse, representing 42 countries located on six continents. This panel contained 1,226 carbapenem-resistant isolates (CRE) [1,027 strains from SENTRY surveillance studies (Tables S2 and S3), including 312 MBL producers (287 NDM, 20 VIM, and 5 IMP) and 199 challenge isolates from Qpex collection with mutations in OmpK36/OmpC including 45 MBL producers] (19) and 521 isolates (from SENTRY surveillance) that carried the most common ESBL genes, such as *bla*_CTX-M_, transferable AmpC, and/or the class D oxacillinases known to hydrolyze broad-spectrum cephalosporins (39).

### Antimicrobial susceptibility testing

Isolates were tested for antimicrobial susceptibility using the broth microdilution methodology per CLSI M07 (2018) guidelines (40). JMI Laboratories produced frozen-form 96-well panels with cation-adjusted Mueller-Hinton broth as the testing medium. Current CLSI quality assurance practices were followed when performing the susceptibility tests. MIC values were validated by concurrently testing the following CLSI-recommended (27) QC reference strains: *E. coli* NCTC 13353 (CTX-M-15), *Klebsiella pneumoniae* ATCC BAA-2814 (KPC-3), *Klebsiella pneumoniae* ATCC BAA-1705 (KPC-2), and *K. pneumoniae* ATCC 700603 (SHV-18). The inoculum density during susceptibility testing was monitored by bacterial colony counts. Where applicable, QC ranges for tested reference strains were those approved or published by the CLSI (27). Meropenem was purchased from Carbosynth; ceftolozane was synthesized at Acme Bioscience, Palo Alto, CA, USA; all other antibiotics were from Sigma Aldrich. Xeruborbactam was synthesized at Qpex Biopharma, San Diego, CA, USA. Avibactam was purchased from eNovation, Chemicals LLC, Bridgewater, NJ, USA. Vaborbactam was synthesized at The Medicines Company, San Diego, CA. Relebactam, taniborbactam, and relebactam were synthesized at Acme Bioscience.

### Rationale for selecting the *in vitro* testing concentration of xeruborbacram

Based on currently available information, *in vitro* activity of meropenem-xeruborbactam is measured as activity of meropenem in the presence of xeruborbactam at a fixed concentration of 8 µg/mL. The rationale for choosing 8 µg/mL of xeruborbactam for *in vitro* testing was based on *in vitro* concentration-response experiments using β-lactamase-producing strains of target pathogens, PK-PD modeling, a safety and pharmacokinetics studies in human volunteers, and correlation of results in non-clinical models of infection.

Concentration of xeruborbactam required to shift at least 90% isolates of CRE (19), CRAB (39), and *P. aeruginosa* to a meropenem MIC of ≤8 µg/mL, the PK-PD susceptibility breakpoint for the high-dose meropenem (41), was ≥4 µg/mL. PK-PD studies on a neutropenic mouse model of infection established that the pharmacodynamics of xeruborbactam administered with a fixed dosage regimen of meropenem (simulated to correspond to the human dose of 2 g administered as 3-hour infusion every 8 hours) was well described by 24-hour free plasma xeruborbactam area under the curve (AUC) and allowed to determine the magnitude of the PK-PD measure associated with 1 log kill targets (16). Phase I clinical studies established a safe human xeruborbactam dosage regimen that meets or exceeds non-clinical PK-PD targets (41). Mouse efficacy of humanized dose regimen of meropenem-xeruborbactam was best correlated with meropenem MICs determined with xeruborbactam at 8 µg/mL: 1 log of bacterial killing was achieved with strains that had meropenem MIC of ≤8 or ≥32 mg/L when tested with xeruborbactam at 8 or 4 µg/mL, respectively. As meropenem is administered using a dosage regimen that simulates 2 g every 8 hours by 3-hour infusion, meropenem can only produce 1 log of bacterial killing with strains that have an MIC of 8 µg/mL or less. With the xeruborbactam testing concentration at 4 µg/mL, the meropenem MICs are ≥32, and 1 log of bacterial killing would not be able to be achieved if these values reflected the *in vivo* situation. When tested with xeruborbactam at 8 µg/mL, the meropenem MICs all fall in a range where 1 log of bacterial killing should be achieved. This supports 8 µg/mL as an *in vitro* testing concentration for xeruborbactam in combination with meropenem. Of note, xeruborbactam steady-state plasma concentration is maintained at a nearly constant level of ~7 µg/mL in phase I subjects following a 500-mg loading dose then 250 mg every 8 hours by 3-hour infusion, providing further support for an *in vitro* testing concentration of 8 µg/mL (41). For several reasons, we have chosen to also perform the testing at 4 µg/mL (i) to have concentration-response data, (ii) for comparative purpose with other combinations that have BLIs tested at 4 µg/mL, and (iii) to obtain MIC for xeruborbactam combinations for strains that are inhibited by xeruborbactam at 8 µg/mL (28).

### Assessment of the relative uptake of BLIs

The relative uptake of BLIs was assessed based on the ratio between IC_50_ of a β-lactamase (NDM-1, VIM-1, or KPC-2) inhibition in whole cells vs IC_50_ of a β-lactamase in cell lysates. The higher the ratio, the lower is the rate of uptake of a BLI across the outer membrane.

#### Determination of IC_50_ of β-lactamase inhibition in whole cells of β-lactamase-producing strains

Cells of *K. pneumoniae* producing NDM-1 (KP1081) or VIM-1 (KP1015) or *A. baumannii* producing KPC-2 (AB1291) were grown in Luria-Bertani (LB) media for 3–5 hours to OD_600_ = 0.7–0.9. Cells were spun down, washed twice in half of the original culture volume of 50-mM Na-phosphate, pH 7.0, 0.5% glucose, 1-mM MgCl_2_ (buffer A), and resuspended in 1/10th of the original culture volume in the same buffer. Cell density was measured and adjusted to OD_600_ = 2 using the same buffer. Bacterial suspension of 50 µL was mixed in triplicates with 50 µL of buffer A, 50 L of 4× concentrated solution of imipenem in buffer A (final concentration 200 or 400 µM for the MBLs or KPC-2-producing strains, respectively), and 50 µL of 4× concentrated BLIs solution in buffer A (final concentration ranging from 500 to 0.0085 µM) and transferred to a 96-well plate, and absorbance profiles at 294 nm were immediately recorded every 30 seconds for 1 hour using SpectraMax plate reader. Initial reaction rates were calculated in optical density (OD)/minute and used to generate dose-response curves vs BLI concentration. IC_50_ values of BLI effect on IMP hydrolysis in whole cells were calculated by fitting the resulting curves in “dose-response – inhibition, variable slope (four parameters)” equation using Prizm software.

#### Bacterial lysate preparation and determination of IC_50_ for BLIs

Same cell suspensions of β-lactamase-producing strains that were grown in LB media to OD_600_ = 0.7–0.9 were subjected to four cycles of 1-minute ultrasonication with 5-minute pause on ice. Resulting lysate (50 µL) was mixed in triplicates with 50 µL of 50 mM Na-phosphate pH7.0, 0.1 mg/mL BSA (buffer B), 50 µL of 4× concentrated solution of imipenem (final concentration 200 or 400 µM for the MBLs or KPC-2-producing strains, respectively) in buffer B and 50 µL of 4× concentrated BLI solution in buffer B (final concentration ranging from 500 to 0.0085 µM), transferred to a 96-well plate, and absorbance profiles at 294 nm were immediately recorded every 10 seconds for 30 minutes using SpectraMax plate reader. Initial reaction rates were calculated in OD/minute and used to generate dose-response curves vs BLI concentration. IC_50_ values of BLI effect on imipenem cleavage were calculated by fitting the resulting curves in the equation dose-response – inhibition, variable slope (four parameters) using Prizm software.

### Statistical analysis

All kinetic results are presented as average ± standard deviation of a minimum of three replicates.

## SUPPLEMENTAL MATERIAL

Supplemental Tables and Figure aac.00440-23-s0001.pdf Several Tables and one Figure.

ASM does not own the copyrights to Supplemental Material that may be linked to, or accessed through, an article. The authors have granted ASM a non-exclusive, world-wide license to publish the Supplemental Material files. Please contact the corresponding author directly for reuse.
