# Supplementary material for: In vitro potency of xeruborbactam in combination with multiple β-lactam antibiotics in comparison with other β-lactam/β-lactamase inhibitor (BLI) combinations against carbapenem-resistant and extended-spectrum β-lactamase-producing Enterobacterales
Source: Antimicrob Agents Chemother. 2023 Oct 6;67(11):e00440-23. doi: 10.1128/aac.00440-23 (PMC10648875; doi:10.1128/aac.00440-23)

Supplementary Tables

Table S1. In vitro potency of xeruborbactam and taniborbactam combinations against CLSI approved quality control strains

Reference strain/antimicrobial agents	MIC (µg/ml) (number of observations)	CLSI approved MIC range (µg/ml)
***Escherichia coli* NCTC 13353 (CTX-M-15)**		
Cefepime	32 (1)-64(5)->128 (10)	>64
Cefepime-taniborbactam (4)	0.125 (13) - 0.25 (4)	0.125/4-1/4
Cefepime-xeruborbactam (4)	≤0.03 (16)	NA
Cefepime-xeruborbactam (8)	≤0.03 (16)	NA
Xeruborbactam	4 (1) - 8 (8) - 16 (2)	NA
***Klebsiella pneumoniae* ATCC 700603 (SHV-18)**		
Cefepime	0.5(1)-1(8)-2(1)-4(1)	0.5-2
Cefepime-taniborbactam (4)	0.25 (11)	0.125/4-0.5/4
Cefepime-xeruborbactam (4)	≤0.03 (5)-0.06 (5)-0.125 (1)	NA
Cefepime-xeruborbactam (8)	≤0.03 (10)-0.06 (1)	NA
Xeruborbactam	8 (7)-16 (4)	NA
***Klebsiella pneumoniae* ATCC BAA-1705 (KPC-2)**		
Cefepime	32 (40)->32(6)	NA
Cefepime-taniborbactam (4)	0.125 (26)-0.25 (14)- 0.5 (6)	0.125/4-1/4
Cefepime-xeruborbactam (4)	0.03 (8)-0.06 (36)-0.125 (4)	NA
Cefepime-xeruborbactam (8)	<=0.015 (22)-0.03 (20)-0.06 (2)-0.125 (2)	NA
Meropenem	8 (2)-16 (16)-32 (28)	8.0-64
Meropenem-xeruborbactam (4)	<=0.015 (46)	NA
Meropenem-xeruborbactam (8)	<=0.015 (46)	NA
Xeruborbactam	8 (2)-16 (44)-32 (2)	NA
***Klebsiella pneumoniae* ATCC BAA-2814 (KPC-3)**		
Cefepime	>32 (61)	NA
Cefepime-taniborbactam (4)	0.5 (16) - 1 (43)-2 (2)	NA
Cefepime-xeruborbactam (4)	0.125 (8)-0.25 (54)-0.5 (3)	NA
Cefepime-xeruborbactam (8)	<=0.03 (8)-0.06 (8)-0.125 (38)-0.5 (2)	NA
Meropenem	>32 (56)	32-256
Meropenem-xeruborbactam (4)	≤0.03 (4)-0.06 (8)-0.125 (44)	0.03/4-0.125/4
Meropenem-xeruborbactam (8)	≤0.03 (28)-0.06 (48)	0.015/8-0.06/8
Xeruborbactam	16 (20)-32 (28)	NA

***Pseudomonas aeruginosa* ATCC BAA-3197 (KPC-2)**		
Cefepime	>128 (16)	NA
Cefepime-taniborbactam (4)	4(1)-8 (15)	NA
Cefepime-xeruborbactam (4)	4 (15)-8 (1)	NA
Cefepime-xeruborbactam (8)	4 (16)	NA
Meropenem	>128 (14)	128-1024
Meropenem-xeruborbactam (4)	2(12)-4(1)-8(1)	NA
Meropenem-xeruborbactam (8)	1 (3)-2(10)-4(1)	1/8-4/8
Xeruborbactam	64 (1)- >128 (13)	NA

Table S2. Carbapenem-resistant surveillance isolates of *Enterobacterales* used in this study*

Organism/ beta-lactamase type		Serine beta-lactamases				Metallo beta-lactamases		
	Total	KPC	Other class A	OXA-48-like	Non-CP CRE	NDM	VIM	IMP
Citrobacter amalonaticus / farmeri	1	1						
Citrobacter freundii species complex	7	3		2	1	1		
Enterobacter aerogenes	11	3			8			
Enterobacter cloacae species complex	85	26	1 (IMI)	8	10	34	6	
Enterobacter hormaechei	2	1				1		
Escherichia coli	55	12		7	11	24	1	
Klebsiella aerogenes	4				4			
Klebsiella oxytoca	26	14			1	9	1	1
Klebsiella pneumoniae	806	297	2	217	63	214**	12**	1
Proteus mirabilis	6				1	3		2
Providencia stuartii	1						1	
Raoultella ornithinolytica	1	1						
Serratia marcescens	22	3	5 (SME-4)	13		1		
Total	1027	261	8	247	99	287	21	4

* The majority of strains that produced serine carbapenemases or metallo beta-lactamases also co-produced other beta-lactamases.

**Eight NDM- and five VIM-producing strains of *K. pneumoniae* also produced KPC beta-lactamase; one strain of *K. pneumoniae* produced both NDM and VIM MBLs.

Table S3. Carbapenemases identified in the surveillance panel of *Enterobacterales*

Class A type	Number	Class D type	Number	NDM type	Number	VIM type	Number	IMP type	Number
KPC-2	192	OXA-48	217	NDM-1	222	VIM-1	17	IMP-1	1
KPC-3	168	OXA-163	1	NDM-4	7	VIM-12	1	IMP-4	1
KPC-4	1	OXA-181	4	NDM-5	33	VIM-19	3	IMP-27	1
KPC-6	2	OXA-232	25	NDM-7	23			IMP-64	1
IMI	1			NDM-9	2				
SME-2	1								
SME-4	4								
Total	369		247		287		21		4

Table S4. In vitro potencies of meropenem alone and combined with xeruborbactam at 4 µg/ml and 8 µg/ml and comparator BLI combination agents against surveillance carbapenem-resistant strains of *Enterobacterales* with xeruborbactam MIC > 8 µg/mL by the carbapenemase present

	MEM	MEM+XER at 4 µg/ml	MEM+XER at 8 µg/ml	FEP	FEP+TAN at 4 µg/ml	CAZ+AVI at 4 µg/ml
ALL	N=891					
MIC_{50}	32	0.06	≤0.03	>32	1	1
MIC_{90}	>32	0.5	0.25	>32	8	32
no MBL	N=634					
MIC_{50}	16	0.06	≤0.03	>32	1	1
MIC_{90}	>32	0.25	0.25	>32	4	2
KPC	N=307					
MIC_{50}	32	0.06	≤0.03	>32	0.25	1
MIC_{90}	>32	0.125	0.125	>32	2	4
OXA-48	N=231					
MIC_{50}	32	0.06	0.06	>32	1	1
MIC_{90}	>32	0.125	0.125	>32	4	2
non-CP CRE	N=96					
MIC_{50}	8	0.25	0.125	>32	2	1
MIC_{90}	16	1	0.5	>32	8	4
MBL	N=257					
MIC_{50}	>32	0.06	≤0.03	>32	2	>32
MIC_{90}	>32	8	2	>32	32	>32
NDM	N=232					
MIC_{50}	>32	0.06	≤0.03	>32	2	>32
MIC_{90}	>32	8	2	>32	32	>32
VIM	N=20					
MIC_{50}	>32	≤0.03	≤0.03	>32	0.5	>32
MIC_{90}	>32	0.5	0.06	>32	4	>32

Abbreviations: MEM, meropenem; FEP, cefepime; CAZ, ceftazidime; XER, xeruborbactam; TAN, taniborbactam; AVI, avibactam

FIGURES

Supplementary Figure S1. Distribution of Xeruborbactam MIC in Carbapenem-Resistant *Enterobacterales* (N=1027) (A) and meropenem-xeruborbactam (4 µg/mL) MIC in strains with xeruborbactam MIC ≤ 8 µg/mL (N=136; 13.3%) (B)

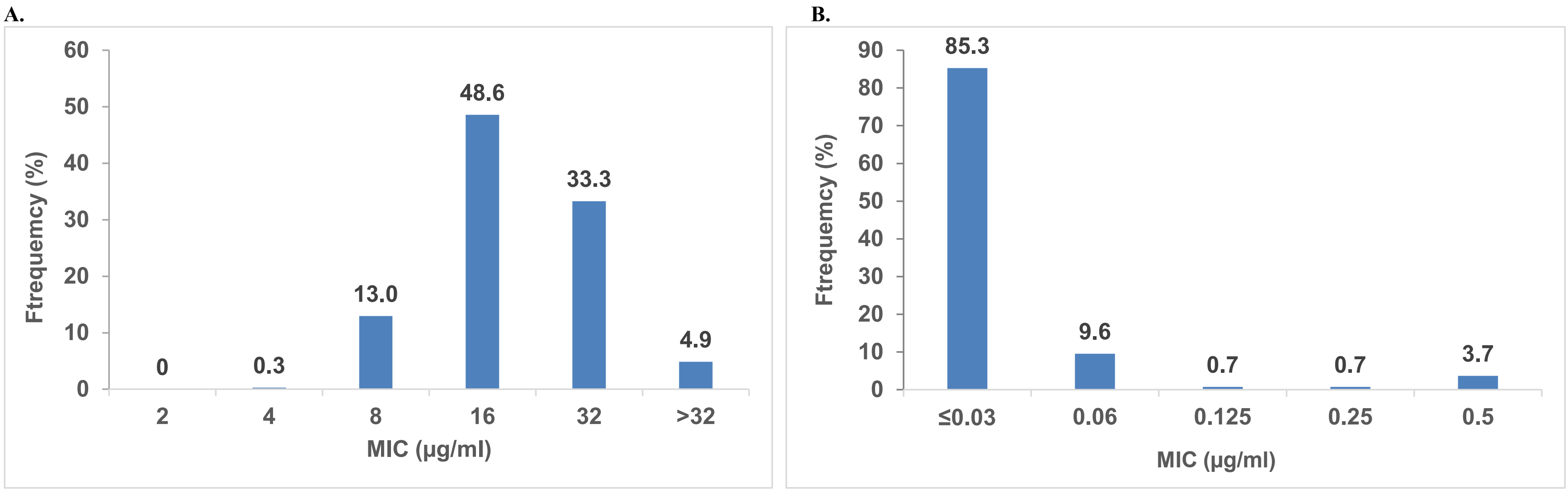

Supplement: Supplemental Tables and Figure — Several Tables and one Figure. [file aac.00440-23-s0001.pdf]
